# Long-term prognosis of symptomatic isolated middle cerebral artery disease in Korean stroke patients

**DOI:** 10.1186/1471-2377-11-138

**Published:** 2011-11-04

**Authors:** Mi Sun Oh, Kyung-Ho Yu, Min-Kyung Chu, Hyeo-Il Ma, Yun Joong Kim, Joo Yong Kim, Byung-Chul Lee

**Affiliations:** 1 Department of Neurology, Hallym University Sacred Heart Hospital, Hallym University College of Medicine, Anyang, South Korea

## Abstract

**Background:**

This study aimed to investigate the long-term mortality and recurrence rate of stroke in first-time stroke patients with symptomatic isolated middle cerebral artery disease (MCAD) under medical management.

**Methods:**

We identified 141 first ever stroke patients (mean age, 64.4 ± 12.5 years; 53% male) with symptomatic isolated MCAD. MCAD was defined as significant stenosis of more than 50% or occlusion of the MCA as revealed by MR angiography. The median follow-up was 27.7 months. We determined a cumulative rate of stroke recurrence and mortality by Kaplan-Meier survival analyses and sought predictors using the Cox proportional hazard model.

**Results:**

The cumulative composite outcome rate (stroke recurrence or any-cause death) was 14%, 19%, 22%, and 28% at years 1, 2, 3, and 5, respectively. The annual recurrence rate of stroke was 4.1%. The presence of diabetes mellitus was the only significant independent predictor of stroke recurrence or any cause of death in multivariate analyses of Cox proportional hazard model adjusted for any plausible potential confounding factors.

**Conclusions:**

We estimated the long-term prognosis of stroke patients with isolated symptomatic MCAD under current medical management in Korea. Diabetes mellitus was found to be a significant predictor for stroke recurrence and mortality.

## Background

Intracranial arterial disease is an important cause of ischemic stroke, especially in Asian populations. According to previous studies, the prevalence of intracranial arterial disease is approximately 8-10% in Caucasian stroke patients [1-3]. In Asian stroke patients, the prevalence ranges from 31% up to as high as 50% [4-7], and middle cerebral artery stenosis is the most common vascular lesion [8]. In the Korean stroke registry, which is a prospective multicenter hospital-based stroke databank compiled by 26 university hospitals in Korea, large artery atherosclerosis accounted for 36% of all ischemic stroke subtypes [9]. Vascular lesions, based on magnetic resonance angiography (MRA), were most commonly found in the middle cerebral artery in 34.8% of patients [10].

Despite the high prevalence of middle cerebral artery disease (MCAD), the natural history and prognosis of stroke patients with MCAD remain unknown. Previous studies showed that patients with MCAD were at a high risk of stroke recurrence [11-13], and the annual stroke risk was between 4% and 15%, even though the patients received medical management [14]. These reports, however, had some limitations because they did not exclude stroke patients with either ipsilateral carotid artery stenosis or a cardioembolic source. Very few reports have focused on the natural history and prognosis of stroke patients with isolated MCAD. Recently, intracranial angioplasty/stenting has been proposed as a promising treatment for patients with cerebral ischemic events [15,16]. However, this procedure has been associated with a short-term morbidity rate of 10-33.3%, a mortality rate of 2.5-8.3%, and a re-stenosis rate of 0-50% after a few months of follow-up [17-19]. Therefore, knowledge of the natural course of MCAD and the effect of current optimal medical therapy is necessary.

The objective of this study was to investigate the long-term stroke recurrence rate and mortality of symptomatic isolated MCAD managed by current medical therapy. We also attempted to identify potential predictors for stroke recurrence and mortality in these patients.

## Methods

Between March 2002 and December 2007, 2,254 patients were admitted to Hallym University Sacred Heart Hospital within seven days of the onset of symptoms of an acute ischemic stroke or transient ischemic attack (TIA). We selected patients with neurological deficits correlated with ischemic events in the MCA territory and in whom MRA showed definitive evidence of MCAD. Patients with a cardiogenic embolic source or non-atherosclerotic causes of ischemic stroke, such as vasculitis, vasospasm, dissection, aneurysm, or Moyamoya disease, or with evidence of recurrent stroke were excluded. We also excluded patients with significant stenosis of extracranial or other major intracranial arteries.

We defined MCAD as the presence of a significant stenosis of more than 50% or occlusion as revealed by MRA (Philips Gyroscan Intera, 1.5 T, 3D TOF MR: 35/9.6 TR/TE, flip angle 25°, 256 × 512 matrix size, 200-mm field of view, 32-mm slab thickness). When MRA reports did not specify the percentage of stenosis, descriptive terms, such as "severe stenosis", "high- or moderate-grade stenosis", or "flow gap" (discontinuity of the blood flow column signal with distal reconstitution), were accepted as evidence of more than 50% stenosis. For all cases with complete MCA occlusion, we performed extensive etiological work-ups, including transesophageal echocardiography and 24-hour holter monitoring, to rule out the possibility of an embolic origin. We additionally repeated transcrainal doppler to identify the recanalization or persistence of MCA occlusion before discharge.

We reviewed the medical records of patients and determined the demographic characteristics, risk factors, and medical management during the follow-up period, and timing of new stroke events or death. However, for patients who had been referred to primary care physicians at the time of discharge, we conducted a telephone interview to assess their compliance with the management for the prevention of secondary stroke and the occurrence of any new stroke events or mortality.

Stroke recurrence was defined as clinical manifestations of neurological deficits correlated with a new lesion in CT or MRI brain imaging. The composite outcome of stroke recurrence or any-cause death was evaluated during the follow-up period to elucidate the long-term prognosis of symptomatic isolated MCAD.

The study protocol was approved by local ethics committee of Hallym University Sacred Heat Hospital.

### Statistical Analyses

In the statistical analysis, a Kaplan-Meier curve was generated for the cumulative probabilities of stroke recurrence and composite outcome (stroke recurrence or any-cause death). We used the Cox proportional hazard regression for univariate and multivariate analyses to identify potential predictors of stroke recurrence or any-cause death. Patients lost for follow-up were censored at the last contact date in the analyses. All data were analyzed using SPSS (version 10.0) software.

## Results

During the study period, 520 (23.0% of the total) acute stroke patients with MCAD were admitted to our hospital. Among them, 180 patients were diagnosed to have significant MCAD as a unique cause of TIA or stroke, and 141 with first-time stroke with symptomatic isolated MCAD were chosen as the final study subjects.

The mean age was 64.4 ± 12.5 years, and 53% of the patients were male. The distribution of the baseline characteristics is listed in Table 1. As a qualifying event, 130 (92.2%) patients had ischemic stroke and 11 (7.8%) had TIA. The mean NIHSS score at the time of admission was 6.1 (range 0 to 25; median 4.0), and the mean modified Rankin scale at discharge was 2.1 (range 0 to 5; median 2.0). Ninety-eight patients had symptomatic isolated MCA stenosis, and 43 patients had complete occlusion of the MCA.

**Table 1:** The comparison of baseline characteristics between the patients with symptomatic isolated MCA disease (IMCAD) and those without.

	With symptomatic IMCAD (N = 141)	Without symptomatic IMCAD (N = 2,113)
Age - mean (SD), years	64.4 (12.5)	66.1 (12.3)
Male sex (%)	74 (52.5)	1,145 (54.2)
Hypertension - no. (%)	87 (61.7)	1,417 (67.1)
Diabetes mellitus - no. (%)	40 (28.4)	664 (31.4)
Smoking (ever) - no. (%)	46 (32.6)	648 (30.7)
Dyslipidemia - no. (%)	53 (37.6) *	419 (19.8)
Time from stroke onset to admission		
≤ 24 hours - no. (%)	78 (55.3)	1,230 (58.2)
> 24 hours - no (%)	63 (44.7)	883 (41.8)
Qualifying event		
Stroke - no. (%)	130 (92.2)*	2,208 (96.0)
TIA - no. (%)	11 (7.8)	85 (4.0)
NIHSS on admission - mean (SD)	6.1 (6.0)	5.8 (6.1)
Median	4.0	3.0
mRS on discharge - mean (SD)	2.1 (1.5)	2.0 (1.6)
median	2.0	2.0
IMCAD		
≥ 50% MCA stenosis	98 (69.5)	
MCA occlusion	43 (30.5)	

IMCAD = Isolated Middle Cerebral Artery Disease; SD = Standard Deviation; TIA = Transient Ischemic Attack

* P < 0.05

The median follow-up was 27.7 months. Sixty-six (47%) patients were treated at our hospital for secondary prevention, and 31 (22%) patients were referred to primary care physicians after discharge. We were unable to follow-up with 28 (19%) patients. Sixteen (11.3%) patients died during the follow-up periods. The cause of death was classified as ischemic (n = 2) or hemorrhagic (n = 1) stroke in three patients, 1 died from ischemic heart disease, 2 from sudden death, 3 from cancer, 4 from pulmonary causes (pneumonia or pulmonary embolism), and 3 from miscellaneous causes. At the last follow-up, all patients were put on antithrombotics, such as aspirin, clopidogrel, or cilostazol, and had appropriate management for risk factors: of 141 patients with symptomatic isolated MCAD, 67% were on anti-hypertensive medication, 31.2% were on anti-diabetic medication, and 47.2% were on lipid-lowering agent.

**Figure 1:**
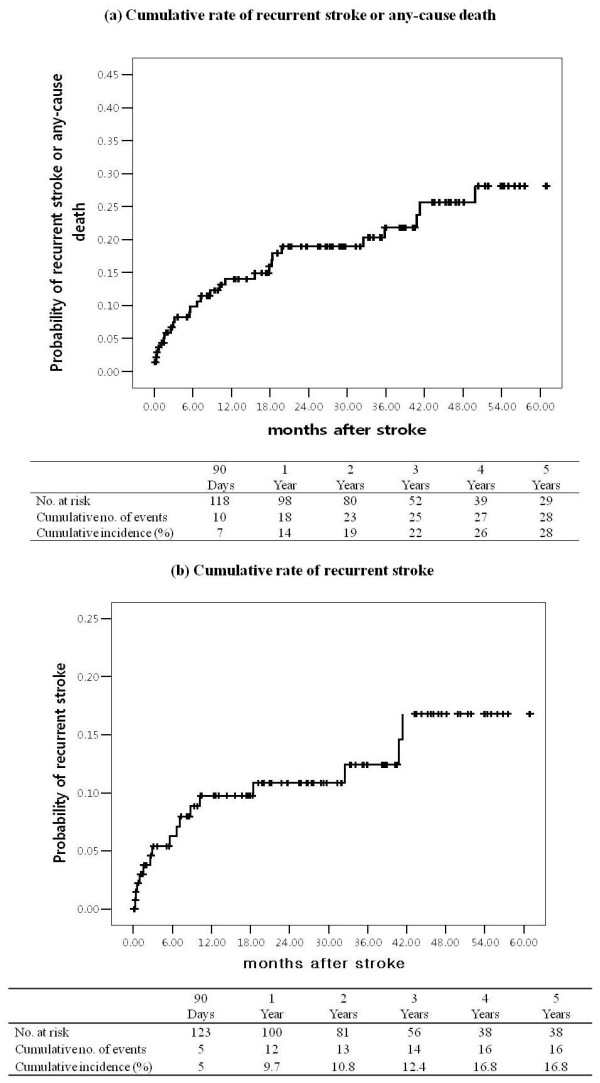
**The Kaplan-Meier curves show (a) the cumulative probabilities of stroke recurrence or any-cause death and (b) stroke recurrence during the observation period**.

During the follow-up period, 29 composite outcomes were documented. The cumulative stroke recurrence or any-cause death rate was 14% at one year, 19% at two years, 22% at three years, and 26% at four years, and the five-year cumulative recurrence of stroke or death rate was 28% in the Kaplan-Meier plots (Figure 1(a)).

Stroke recurrence was noted in 16 patients, resulting in an overall annual rate of 4.1%. Eleven events (68.7%) occurred in the vascular territory of symptomatic ipsilateral MCAD. Stroke out of the territory of the symptomatic MCAD occurred in 5 (31.3%) of the 16 patients. All events were ischemic stroke. Stroke events ipsilateral to the MCAD occurred at a mean of 10.4 months after the qualifying event, and recurrent stroke in remote or other territories occurred after 14.5 months.

Using the Kaplan-Meier method (Figure 1(b)), the cumulative stroke recurrence rate was estimated to be 9.7% at year one, 10.8% at year two, 12.4% at year three, and 16.8% at year five.

**Figure 2:**
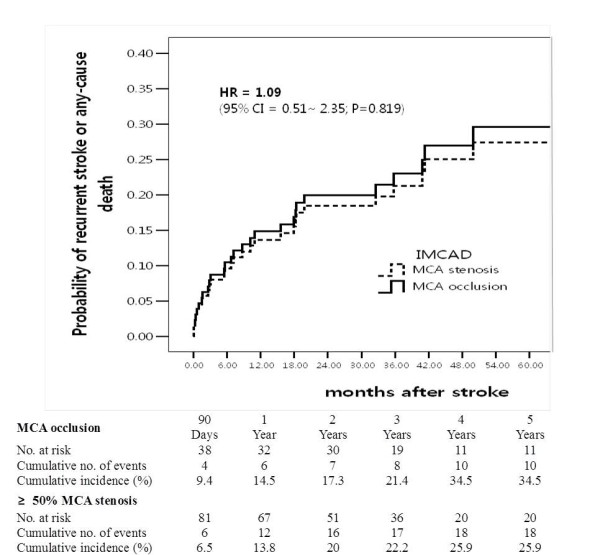
**The Kaplan-Meier curves show the cumulative rate of recurrent stroke or any cause of death during the observation period**. Comparison of the patients with occlusion (upper curve) and with ≥ 50% stenosis (lower curve).

No significant difference between MCA stenosis and MCA occlusion was noted for the cumulative probabilities of stroke recurrence or any cause of death (Figure 2).

In the univariate analyses conducted to identify the potential predictors for the composite outcome, the presence of diabetes mellitus was the only significant factor (HR = 2.16, 95% CI = 1.04-4.49) (Table 2). In the multivariate analyses of the Cox proportional hazard model adjusted for any plausible potential confounding factors such as age, sex, MCA occlusion vs. stenosis, and the time from stroke onset to admission, which were associated with the prognosis of MCAD in a previous study[7,20], the presence of diabetes mellitus was significant as an independent predictor of the composite outcome (Table 3).

**Table 2:** Potential predictors of stroke recurrence or any-cause death in the univariate analyses

	Hazard Ratio (HR) * **(95% CI)**	P value
Age (≥ 64 vs. < 64)	1.97 (0.87-4.44)	0.105
Sex (male vs. female)	1.13 (0.55-2.36)	0.738
Hypertension (yes vs. no)	1.22 (0.56-2.69)	0.619
Diabetes mellitus (yes vs. no)	2.16 (1.04-4.49)	0.039
Dyslipidemia (yes vs. no)	0.97 (0.46-4.05)	0.940
Smoking (yes vs. no)	0.74 (0.33-1.70)	0.465
Time from stroke onset to admission (> 24 hours vs. ≤ 24 hours)	1.57 (0.73-3.39)	0.248
Qualifying event (Stroke vs. TIA)	1.22 (0.29-5.15)	0.783
NIHSS at admission (≥ 4 vs. < 4)	1.72 (0.75-3.91)	0.198
mRS at discharge (2-5 vs. 0-1)	2.06 (0.84-5.09)	0.117
IMCAD (occlusion vs. ≥ 50% stenosis)	1.09 (0.51-2.35)	0.819
		

* The hazard ratio and HR p-value of the each variable were determined with the use of a Cox proportional-hazards regression model.

**Table 3:** Multivariate analyses of the Cox proportional hazard model of DM associated with stroke recurrence or any-cause death, adjusted for age, sex, MCA stenosis vs. occlusion, and the time from stroke onset to admission.

	Adjusted Hazard Ratio (95% CI)	P-value
Age (≥ 64 vs. < 64)	1.97 (0.87-4.50)	0.106
Sex (male vs. female)	0.98 (0.46-2.07)	0. 958
IMCAD (occlusion vs. ≥ 50% stenosis)	1.31 (0.60-2.87)	0.498
Time from stroke onset from admission (> 24 hours vs. ≤ 24 hours)	1.83 (0.84-4.02)	0.130
Diabetes Mellitus (yes vs. no)	2.26 (1.07-4.77)	0.032

IMCAD = Isolated Middle Cerebral Artery Disease; TIA = Transient Ischemic Attack

## Discussion

We used MRA to verify 141 first-time ischemic stroke patients with symptomatic isolated MCAD and analyzed the long-term prognosis of stroke recurrence rate and mortality for isolated MCAD. The one-year cumulative stroke recurrence or any-cause death rate was 14%, and the five-year cumulative stroke recurrence or death rate was 28%. The annual recurrence rate was 4.1%, and the cumulative rate was 9.7% at year one and 16.8% at year five in patients with isolated MCAD under the current standard medical treatment, including antithrombotics and strict control of risk factors such as hypertension and diabetes mellitus, statins for hyperlipidemia, and smoking cessation. As an independent predictor of stroke recurrence or any-cause death, diabetes mellitus was significant.

The prognosis of patients with MCAD under conservative medical therapy has varied among different studies because they used different diagnostic tools, and MCAD may occur separately or in conjunction with carotid artery diseases [21,22]. Previous studies have reported that the recurrence rate of stroke is 2-24%, and the mortality rate is 0-29% in patients with MCAD [21,23-25]. In our study, the overall annual rate of stroke recurrence was 4.1%, which appeared to be more favorable than the results of a previous study, in which cerebrovascular events (TIA and stroke) occurred in 11.7% of the patients per year during follow-up (42 months) of 164 medically treated patients enrolled in the Extracranial/Intracranial Bypass Study [26].

The previous studies reported that the rate of stroke recurrence was similar in patients with severe MCA stenosis and MCA occlusion [27-30]. Likewise, we also found that the long-term prognosis, stroke recurrence and any-cause death in patients with MCA occlusion did not differ from patients with MCA stenosis.

Patients with symptomatic MCAD are regarded as being at high risk for recurrent cerebrovascular events [11,14,27,31,32]. The cumulative recurrence rate of symptomatic MCAD seems to be even higher than that of symptomatic extracranial carotid artery disease [33]. Therefore, alternative therapies, such as aggressive management of risk factors, other antiplatelet regimens, and intracranial angioplasty/stenting, are needed. Intracranial angioplasty and stenting has been explored as a promising treatment for patients with symptomatic intracranial stenosis [15,16]. However, the first prospective randomized study, stenting versus aggressive medical management for preventing recurrent stroke in intracranial stensosis (SAMMPRIS), has very recently been halted because high complication rate in the stenting arm (14% patients had a stroke or died in the 30-day period after stenting compared to only 5.8% in the medical arm) [34]. Although the medical management in order to achieve target levels based on national guidelines during the follow-up was identical in the two groups, there were significantly differences between two groups with respect to some of the measures of risk factors at various times during the trial [34].

Current medical treatments for secondary prevention of stroke mainly consist of risk factor control and antithrombotic therapy. Whether anticoagulation is superior to antiplatelet therapy for patients with symptomatic intracranial artery stenosis remains controversial. However, the Warfarin-Aspirin Symptomatic Intracranial Disease (WASID) trial showed that warfarin was not more effective than aspirin in the prevention of recurrent stroke and carried a higher risk of serious bleeding and death [28]. Recently, statins have been shown to lead to regression of atherosclerosis, and one trial showed a reduction in the risk of stroke [35,36]. Poor control of blood pressure and cholesterol appear to be associated with a higher risk of a subsequent stroke [37,38]. However, previous studies have shown that only 35-47% of patients are treated to target after a vascular event [38,39]. In our study, all patients continuously used antithrombotics, more than half used antihypertensive (ACEI or ARB), and more than one third used statin. The finding that the long-term prognosis of symptomatic MCAD in our study appeared to be more favorable than a previously published study may be attributable to a better compliance with antithrombotics and pharmacological therapy of risk factors.

With regard to prediction factors, there is relatively little information on the relationship between risk factors and the recurrence of vascular events in patients with intracranial artery disease. One study identified several variables that were associated with an increased occurrence of ipsilateral stroke in the territory of symptomatic intracranial stenosis in multivariate analyses [20]. In the study, severe stenosis, stroke symptoms, and being a woman were predictors of ischemic stroke. Another study in a Chinese population identified DM as a risk factor for recurrent vascular events or death in a group of patients with predominantly intracranial atherosclerosis [40]. We also indentified the presence of diabetes mellitus as an independent potential predictor of long-term outcome, stroke recurrence, or any-cause death. The progression of MCA stenosis detected by TCD was the only factor independently associated with ipsilateral cerebral ischemic events during follow-up [27,41].

Some limitations of our study should be noted. First, because our study was hospital-based, it has the potential for a selection bias. Therefore, our results may not be representative of the long-term prognosis of MCAD in Korea. Second, we relied on MRA findings to define MCAD rather than conventional angiography. In addition, MRA could overestimate the degree of stenosis. Third, the rate of follow-up loss was 19%. However, there were no significant differences in the baseline characteristics and any-cause death between patients lost to follow-up and those who completed the follow-up (Table 4). The deaths of patients who were lost to follow-up were verified by the information on death source data from the Korean National Statistical Office for the follow-up period. Therefore, the 19% lost to follow-up rate may not have affected the overall clinical outcomes. Finally, since some cases were investigated through telephone interviews for stroke recurrence, minor strokes might have been unnoticed.

**Table 4:** The comparisons of baseline characteristics and any-cause death during follow-up period between the completed follow-up patients and lost to follow-up patients.

	IMCAD (N = 141)	Completed follow-up (n = 113)	Lost follow-up (n = 28)	P
Age - mean (SD), years	64.4 (12.5)	64.7 (12.0)	63.1 (14.4)	0.558
Male sex (%)	74 (52.5)	61 (54.0)	13 (46.4)	0.474
Hypertension - no. (%)	87 (61.7)	73 (64.6)	14 (50.0)	0.155
Diabetes mellitus - no. (%)	40 (28.4)	29 (25.7)	11 (39.3)	0.152
Smoking (ever) - no. (%)	46 (32.6)	39 (34.5)	7 (25.0)	0.336
Dyslipidemia - no. (%)	53 (37.6)	41 (36.3)	12 (42.9)	0.520
Time from stroke onset to admission				0.527
≦24 hours -no. (%)	78 (55.3)	64 (56.6)	14 (50.0)	
> 24 hours-no (%)	63 (44.7)	49 (43.4)	14 (50.0)	
Qualifying event				0.457
Stroke - no. (%)	130 (92.2)	105 (92.9)	25 (89.3)	
TIA - no. (%)	11 (7.8)	8 (7.1)	3 (10.7)	
NIHSS on admission - mean (SD)	6.1 (6.0)	6.3(6.3)	5.3 (4.8)	0.420
mRS on discharge - mean (SD)	2.1 (1.5)	2.1 (1.6)	1.9 (1.4)	0.671
IMCAD				0.805
MCA stenosis ≥ 50%	98 (69.5)	78 (69.0)	20 (71.4)	
MCA occlusion	43 (30.5)	35 (31.0)	8 (28.6)	
Any-cause Death	20 (14.2)	16 (14.2)	4 (14.3)	1.00

## Conclusions

We estimated the long-term prognosis of stroke patients with isolated symptomatic MCAD under current medical management in Korea. Diabetes mellitus was found to be a significant predictor for stroke recurrence or any cause of death.

## Conflicts of Interest Disclosure

The authors declare that they have no competing interests.

## Authors' contributions

MS Oh participated in the study design, performed the statistical analyses, and drafted the manuscript. KH Yu and BC Lee participated in the design of study and the enrollment of patients and helped to draft the manuscript. MK Chu, HI Ma, YJ Kim, and JY Kim participated in the enrollment of patients and reviewed the manuscript.

And all authors read and approved the final manuscript.

## Pre-publication history

The pre-publication history for this paper can be accessed here:

http://www.biomedcentral.com/1471-2377/11/138/prepub
